# Inducing oculomotor plasticity to disclose the functional link between voluntary saccades and endogenous attention deployed perifoveally

**DOI:** 10.1038/s41598-019-54256-1

**Published:** 2019-11-28

**Authors:** Judith Nicolas, Aurélie Bidet-Caulet, Denis Pélisson

**Affiliations:** 1 Integrative Multisensory Perception Action & Cognition Team (ImpAct), Lyon Neuroscience Research Center (CRNL), INSERM U1028, CNRS UMR5292, 69500 Bron, France; 2 Brain Dynamics and Cognition (Dycog Team), Lyon Neuroscience Research Center (CRNL), INSERM U1028, CNRS UMR5292, 69500 Bron, France; 3 University Claude Bernard Lyon 1, Université de Lyon, 69000 Lyon, France

**Keywords:** Attention, Saccades

## Abstract

To what extent oculomotor and attention systems are linked remains strongly debated. Previous studies suggested that saccadic adaptation, a well-studied model of oculomotor plasticity, and orienting of attention rely on overlapping networks in the parietal cortex and can functionally interact. Using a Posner-like paradigm in healthy human subjects, we demonstrate for the first time that saccadic adaptation boosts endogenous attention orienting. Indeed, the discrimination of perifoveal targets benefits more from central cues after backward adaptation of leftward voluntary saccades than after a control saccade task. We propose that the overlap of underlying neural networks actually consists of neuronal populations co-activated by oculomotor plasticity and endogenous attention deployed perifoveally. The functional coupling demonstrated here plaids for conceptual models not belonging to the framework of the premotor theory of attention as the latter has been rejected precisely for this voluntary/endogenous modality. These results also open new perspective for rehabilitation of visuo-attentional deficits.

## Introduction

As much as we would like to, our brain is not able to deal with the huge amount of information brought up by our senses. Especially when it comes to vision, albeit the dominant sense of primates, our brain resources are too limited to efficiently handle visual information sensed by the millions of photoreceptors of our eyes. Therefore, we need to select what part of space we want to pay attention to. Visuospatial attention is a cognitive process which plays a critical role in this selection by facilitating the visual processing of objects and features falling in the area of space where it is focused on, at the expense of those situated outside^1,2^. To get a refined and homogenous analysis of our entire visual field, this attentional focus must be frequently re-oriented either automatically, in response to the sudden appearance of a stimulus (exogenous attention) or voluntarily, being driven by internal goals (endogenous attention)^1^. These two attention-shifting mechanisms are partially distinct, relying respectively on the ventral and dorsal streams of attention^3,4^, and both can either or not be accompanied by eye movements (overt and covert shifts, respectively).

Saccadic eye movements are also of utmost importance to explore our visual environment and select meaningful information therein. Indeed, as visual acuity is highest in the narrow central zone of the visual field processed by the fovea, gaze shifts are mandatory to explore a visual scene. Like attention shifts, gaze shifts are either exogenously or endogenously triggered, corresponding to so-called reactive (RS) or voluntary saccades (VS), respectively. Shifts of attention and saccadic eye movements share several other features, up to the point that, in the framework of the premotor theory of attention, attention shifts are considered to be unexecuted saccades inhibited at the oculomotor output level^5^.

Saccadic adaptation (SA) is a well-studied sensorimotor adaptation process (see^6,7^ for reviews) and therefore constitutes a convenient tool to assess the role of the oculomotor system on spatial attention. Interestingly, in human, the neural substrates of SA and of visuospatial attention overlap. Indeed, the intraparietal sulcus (IPS) has been involved in both adaptation of VS^8,9^ and endogenous attention (see^3^ for review) while the right temporo-parietal-junction (rTPJ) has been involved in adaptation of RS^8,10–12^ and exogenous attention^3^. Moreover, two behavioral studies have suggested that this overlap might have functional consequences: the first reports increased performances in a visual detection task performed after adaptation of RS^13^, and conversely, the second shows that RS adaptation efficiency is increased when subjects are simultaneously engaged in an attention-demanding task directed to the saccade target^14^. Note, however, that the visual detection task used by Habchi *et al*.^13^ did not allow to specifically isolate covert attention shifts from the other cognitive or motor components involved. In addition, and to the best of our knowledge, the coupling between SA and attention has never been investigated in the endogenous modality. As the premotor theory of attention has been challenged for the voluntary/endogenous modality (see^15^ for review), highlighting a functional link between oculomotor plasticity and endogenous attention would have strong theoretical implications.

Therefore, the present study aimed at investigating the coupling between saccades and visuo-spatial attention in the endogenous modality, using a Posner-like paradigm allowing to specifically assess pure covert attention shifts before and after the development of voluntary saccades adaptation.

## Materials and Methods

### Subjects

The experiment adheres to the code of ethics of the World Medical Association – Declaration of Helsinki (2008) and received the approval of the Ethics Committee of INSERM (CEEI - IRB 00003888, n° 16-305). Forty-one subjects provided a written informed consent before performing the tasks and received a compensation for their participation. Among those subjects, four were excluded because they did not show significant saccadic gain modulation in one of the two adaptation exposures and one was excluded because of poor discrimination performances (for details see section Data Analysis). The remaining subjects were all right-handed except one, comprised 17 males and 19 females, with a mean age of 25.5 +/− 4.53 SD (Standard Deviation). Their vision was normal or corrected-to-normal. Criteria of exclusion were: neurological or psychiatric disorders history; cognitive disorders preventing the comprehension of the instructions; severe sleep deprivation during the last 24 hours; consumption of psychotropic drugs, substances, or alcohol during the last 24 hours; participation to other experiments involving sensorimotor adaptation during the last week. After written consents obtained, each subject was assigned pseudo-randomly to one of the six sub-groups of each experiment, corresponding to the 6 possible orders of testing in the three sessions (within-subject design, see General Design section). The number of subjects was determined from a power analysis performed through the G*Power software^16^ and based on parameters established from the literature and from pilot data (see Power analysis in the Supplementary Methods).

### Apparatus, stimuli and procedure

#### Apparatus

Experiments were carried out in a dimly lit room. Subjects were installed in a comfortable position with the head stabilized by a chin-rest, cheekbone rests, and forehead support; they faced a computer screen (1920 × 1080 pixels; 53.5 × 34.5 cm; 144 Hz refresh rate) at 57 cm from their eyes. Experiments are timed based on the 144 Hz refresh rate of the computer display (frame duration approximately 7 ms), therefore all time-intervals reported in the following represent multiple of the frame duration and are rounded to the nearest value in milliseconds. Psychopy^17^, an open-source software, was used for the stimuli presentation and data collection in all different tasks. Movements of the right eye were recorded at a frequency of 1000 Hz using the remote configuration of the EyeLink 1000 infrared eye-tracker (SR research, Canada). Each task started with the calibration of the eye-tracker by asking subjects to fixate a series of 5 targets displayed near the borders and at the center of the screen.

#### General design

**Figure 1:**
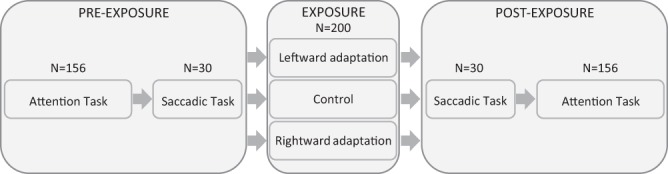
Study general design. In both Experiments 1 and 2, each subject underwent 3 experimental sessions -composed of a pre-exposure, an exposure, and a post-exposure phase - differing only by the exposure phase (either leftward adaptation, rightward adaptation or control). N = number of trials.

Experiments 1 and 2 were carried out separately in two different experimental groups. The two experiments were identical except for the eccentricity of the target in the attention task (see section Attention task). In each experiment, subjects were submitted to three experimental sessions (within-subjects design), each of which (‘leftward adaptation’, ‘rightward adaptation’ and ‘control’) comprising identical pre-exposure and post-exposure phases as well as a specific exposure phase (Fig. 1). During all three exposures, saccades in both directions were performed. In the leftward adaptation, only leftward saccades were adapted; conversely in the rightward adaptation, only rightward saccades were adapted; finally in the control, no saccades were adapted. This control session allowed assessment of unspecific effects of exposure to a saccadic task. The effects on attention were measured by comparing, between the pre- and post-exposure phases of each session, subjects’ performance in a visual discrimination attention task; in addition, comparing the gain of saccades measured during a test saccade task performed before and after exposure allowed us to check for successful saccadic adaptation in the respective hemifields. The delay between each session was at least 14 days in order to avoid any retention of saccadic adaptation between sessions, based on a previous study disclosing a significant retention of adaptation up to 5 days after exposure but not 11 days after^18^.

#### Saccadic tasks

The saccadic adaptation was performed by a modified version of the double-step paradigm introduced by McLaughlin (1967)^19^. This paradigm consists in displacing the visual scene while the subject is executing a saccade towards a peripheral target. Thanks to the saccadic suppression phenomenon, this intra-saccadic visual displacement is usually not consciously perceived by subjects and leads to a mismatch between post-saccadic eye fixation and target location which is interpreted by the central nervous system as a saccade aiming error.

**Figure 2:**
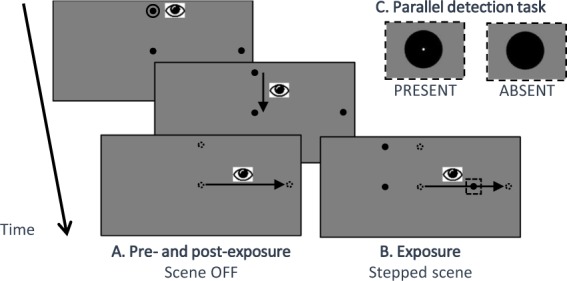
Time-line of a trial in the saccadic tasks (not to scale). After the circle around the fixation point turns off, subjects had to make, at their own pace, a downward saccade to the central point and then a horizontal –voluntary– saccade to the peripheral target. (**A**) In the pre- and post- saccadic phases, the visual scene was turned off as soon as the voluntary saccade was detected. Subjects were instructed to keep looking at the peripheral target position for ~1 sec and then look back to the upper location in anticipation of the fixation point re-appearance, using that time period to blink if necessary. (**B**) In the exposure phase, the visual scene was shifted backward immediately at the voluntary saccade onset (adapted saccades) or after 805 ms (unadapted saccades). The scene remained for 1610 ms in total in both conditions. The size of the shift increased progressively across blocks 1–4 (respectively 1°, 2°, 3° and 3°). (**C**) Enlarged view of peripheral targets during the exposure phase: subjects additionally performed a simple detection task to favor a sustained motivation: they had to report by a push button the presence of a small white dot inside the peripheral target (visible only in perifoveal vision after the saccadic response: see enlarged views of a dot-present target and of a dot-absent target). Feedback regarding this simple detection task was given at the end of each block.

Sequence of events for adapted saccade trials (Fig. 2B): Three dots of 0.3° of visual angle were displayed on the computer screen. The first dot was located 4° above the center of the screen, and was surrounded by a small circle. The second dot was at the center of the screen. The third dot, the peripheral target, was at 9° of eccentricity aligned with the horizontal meridian, either to the left or to the right. The side of the peripheral target was blocked with 12 trials in the adapted direction, 12 in the opposite direction, repeated 2 times for each block. The subject had to fixate the upper dot during a pseudo randomized delay between 301 ms and 701 ms after which the disappearance of the surrounding circle (‘go signal’) indicates that he/she had to look successively at the other two targets. Correct eye fixation of the upper dot was ensured by continuous monitoring of the eye-tracker signal. In the next 2000 ms, the subject had to make at her/his own pace, a first saccade towards the central dot (vertical saccade) and then a second saccade from there towards the peripheral target (horizontal voluntary saccade). The voluntary saccade was detected when the eye velocity was higher than 70°/s^20^. This event immediately triggered the shift of the visual scene when the peripheral target was in the adapted hemifield (Fig. 2B). The visual scene shift was progressively increased through the blocks (1° for the first block, 2° for the second, 3° for the third and fourth blocks) leading to a progressive decrease of the target final eccentricity (8°, 7° and 6° respectively). The visual scene remained visible for a total of 1610 ms after the detection of the voluntary saccade. The subject then had a delay of 1000 ms to blink and look back to the upper dot. The next trial started as soon as correct fixation of the upper dot location was detected.

Sequence of events for unadapted saccade trials: These trials were identical to the adapted saccade trials except that the jump of the visual scene occurred 805 ms after the detection of the voluntary saccade. These trials correspond to the saccades toward the unadapted hemifield for the leftward and rightward exposure and for the saccades toward both hemifields in the control exposure.

The total exposure phase consisted of 196 trials distributed in 4 blocks of 48 trials each (24 with a right target and 24 with a left target). Between each block, the subject was allowed to rest with the head still as long as needed.

To maximize subjects’ involvement and motivation throughout the saccadic tasks, they were requested to perform in parallel an easy detection task: in random trials (from 5 to 20 per block), the peripheral target contained a white dot of 0.008° of visual angle (not detectable in peripheral vision but easy to detect after the saccade to the target), and subjects had to push a button after each trial in which they detected the white dot. Performance feedback was provided to subjects during the rest period between the blocks but was not further analyzed.

Pre- and post-exposure saccadic tasks (Fig. 2A): These tasks were identical to the exposure tasks except that the visual scene did not jump but instead was turned off at the initiation of the voluntary saccade. Each task consisted in one block of 30 trials (15 with a right target and 15 with a left target, randomly ordered). Comparison between pre- and post-exposure tasks allowed determination of the SA after-effect (change of saccade amplitude in post- *versus* pre-exposure) and thus quantitative assessment of the adaptation strength.

#### Attention task: visual discrimination

A variant of the Posner task^1^ was designed with the main features (a central cue, and a long SOA) chosen to evoke shifts of endogenous attention. Contrasting between informative trials (cue always valid) and uninformative trials (uninformative cue) allowed us to measure the pure benefit of endogenous attention orienting. This approach was preferred over that used in many endogenous attentional studies, consisting of contrasting between valid and invalid cues, which rather yields the cumulated effect of exogenous costs and endogenous benefits^21,22^.

**Figure 3:**
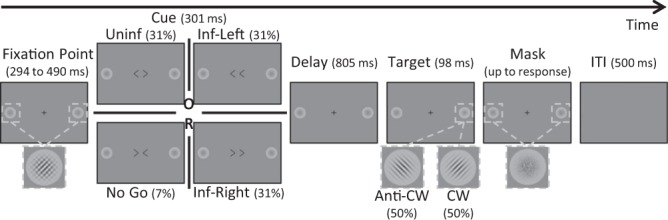
Time-line of a trial in the attention task: A central fixation cross and 2 lateral placeholders (eccentricity: 7.5° in Experiment 1; 3° in Experiment 2) each containing 2 orthogonal gabors, were present at the beginning of the trial. Then central cues appeared for 301 ms, either indicating the side of the upcoming target (100% valid informative cue: Inf-Left or Inf-Right), or providing no spatial information (uninformative cue: Uninf), or indicating to restrain the response (No Go cue). The target presented after 805 ms of delay (SOA = 1106 ms) consists in the brief disappearance of one gabor on one side (left 50% or right 50%), followed after 91 ms by a mask. Using a push/pull device, subjects had to respond as fast and as accurately as possible whether the target was tilted clockwise (CW) or anti-clockwise (anti-CW). Experiments 1 and 2 differed only according to the eccentricity of the discrimination target and associated place-holder.

Sequence of events in the attention task trials (Fig. 3): A fixation cross subtending 1° of visual angle appeared at the center of the screen (grey 50%) at the beginning of the trial and, except during the cue period, remained visible until the subject’s response. Subjects had to keep eye fixation on that location all throughout the trial. Two light grey (35%) placeholders (circles of 2.5° of visual angle in Experiment 1; 1.5° in Experiment 2) were also presented along the horizontal meridian, on the left and on the right, at 7.5° of eccentricity in Experiment 1, and at 3° of eccentricity in Experiment 2. Each placeholder initially contained two gabor patches (Experiment 1: 4 cycles per degree (cpd) of spatial frequency and 2.5° of visual angle; Experiment 2: 4 cpd of spatial frequency and 1.5° of visual angle) presented with a Gaussian mask and superimposed orthogonally (one gabor tilted at 45° and the other at −45° relative to the vertical, leading to the perception of a grid). The contrast of the gabor patches was previously determined for each individual by a staircase procedure to achieve a 80% level of correct discrimination (see Staircase procedure in Supplementary Methods). After a pseudo-randomized (294 to 490 ms) delay from the beginning of the trial, a cue appeared for 301 ms. This cue was composed of two empty arrows (1.5° vertically × 1° horizontally) flanking the center of the screen (1.0° of horizontal spacing). For ~two thirds of the trials (32 ‘informative trials’ out of 52 trials for each block) the cue validly informed the future target location: the two arrows both pointed either toward the left or toward the right of the screen to indicate the placeholder in which the target will appear. In 16 ‘uninformative’ trials (~one third), the cue did not provide any spatial information about the upcoming target, the two arrows pointing outwards. The 1:2 ratio of uninformative versus informative trials was meant to potentiate the cueing effect^23^. In the four remaining trials of each block, a ‘no-go cue’ represented by the two arrows pointing inwards instructed subjects to refrain from answering. These ‘no-go’ trials were meant to enforce subjects to use the cue to perform the task correctly, and thus favoring the conscious interpretation and increasing the benefit of the cue. However, they were not analyzed. In all trials, the cue period was followed first by displaying again the fixation point and then 805 ms after cue offset by a brief extinction (98 ms) of one of the two gabor patches either in the left placeholder (50%) and or in the right placeholder (50%): the remaining gabor patch thus constitutes the target (SOA = 1106 ms) which orientation had to be discriminated. Immediately after this target presentation, a mask was displayed in the two placeholders until the subject’s response was made or for a maximum duration of 1500 ms. Subjects had to discriminate as fast and as accurately as possible whether the target gabor patch was tilted clockwise or anticlockwise (45° or −45° with respect to the vertical, respectively). Subjects answered with their index finger through a double switch device oriented in their mid-sagittal axis, with a response assignment randomized between subjects: half of them pushed the switch for a “clockwise” target and pull it for an “anticlockwise” target, the other half was instructed with the opposite assignment. Eye fixation was continuously monitored all throughout the trial and whenever the subject broke fixation (gaze deviating in any direction more than 1.5° from the fixation cross), the fixation cross immediately turned red and the trial was aborted. Aborted trials were replayed back during the same block of trials. We chose a SOA duration of 1106 ms in order to minimize any involvement of attention oriented exogenously^24^. Moreover, the pilot data reported in Supplementary data showed that the duration of the SOA does not affect the validity effect in our discrimination task.

The task consisted of 3 blocks of 52 trials each (156 in total): 16 ‘informative - left target’ and 16 ‘informative - right target’, 8 ‘uninformative - left target’ and 8 ‘uninformative - right target’, and 4 ‘no-go cue’. Between each block, subjects received standardized feedback about their performance (see Instructions and feedback in Supplementary Methods).

### Data analyses

Data analyses were performed with the open-source software R (The R Core Team, 2013). These analyses concerned the saccadic behavior during the pre- and post-exposure saccadic tasks as well as the performances in the attention tasks measured by cue benefit (relative change of reaction time – RT – between informative and uninformative trials). Any exclusion of a subject due to criteria described in the following paragraphs led to his/her replacement. All the group analyses have been carried out separately for the two experiments.

#### Saccadic tasks

Preprocessing: Eye movement data were analyzed off-line using custom software developed in Matlab (Math Works Inc., Natick, MA, USA). The beginning of the primary horizontal saccade was identified offline based on a velocity threshold of 30°/s. Saccadic amplitude was measured as the difference between eye positions 50 ms before the saccade onset and 50 ms after the saccade offset. The gain of a saccade was used as the dependent variable in the saccadic tasks. It was computed as the ratio between saccadic amplitude and initial target eccentricity (difference between target position and starting position of the saccade). Saccades with a gain less than 0.5 or outside the mean ± 2 SD interval were discarded from further analysis.

Statistical analysis: Since the saccadic adaptation was critical to test our hypothesis, we excluded from the main analysis subjects who did not show the expected decreased gain of saccades in the adapted hemifield. To this aim, we first performed, separately for each subject and each hemifield, a unilateral Student t-test comparing the gain of the saccades between the pre- and the post-saccadic tasks and used a threshold p-value of 0.05 after FDR (False Discovery Rate^25^) correction for 6 multiple comparisons. Moreover, for representational purposes, we computed the exposure after-effect for each hemifield and each exposure condition as follow:

$$Exposure\,after\,effec{t}_{exposureofinterest}=\frac{mean\,gai{n}_{post-exposure}-mean\,gai{n}_{pre-exposure}}{mean\,gai{n}_{pre-exposure}}$$

A negative exposure after-effect reflects a decrease of the saccadic gain between the pre- and the post-exposure phases.

Finally to calculate the effect size of the exposure after-effect in the exposure sessions, we computed the mean of the gain for each subject, in the adapted hemifield for the pre-exposure and the post-exposure phase separately. These values were used to calculate the Cohen’s *d* effect size for Student t-test.

As the results to the attentional task in Experiment 2 revealed a significant effect of leftward backward SA (see section Experiment 2 section of the attentional task in the results), we performed two supplementary analyses on saccadic data of the leftward adaptation exposure.

First, to assess the effect of SA on voluntary saccades preparation time, we computed the fixation time (FT) as the period of time between the end of the first saccade and the beginning of the second (voluntary) saccade. In each subject, we computed the median FT for each saccade direction and each phase of the exposure of interest, namely the backward adaptation of leftward saccades. We then performed a repeated measure ANOVA (rmANOVA) on median FT with the saccade direction (leftward or rightward), and the phase (pre- or post-exposure) as within-factors.

Second, since in Experiment 2, the eccentricity of the discrimination target corresponds to the final size of the intra-saccadic step (ISS, 3°), we searched for any correlation between the cue benefit and the post-saccadic error after leftward, adapted, saccades. Post-saccadic error was measured as the distance between the saccade landing position and the jumped target. The correlations were then computed separately for each block of exposure and one global correlation on post-saccadic error across all blocks.

#### Attention task

Preprocessing: To ensure that the level of involvement of each subject was high, subjects with low global performance or with high fluctuations were excluded. To this aim, each session were divided in 8 experimental cells of conditions (2 cue types × 2 target hemifields × 2 phases, smallest cell = 24 trials). We excluded subjects with a number of correct trials inferior to 8 for any of these cells. Then, trials with outlier RT were excluded using the John Tukey’s method of leveraging the Interquartile Range, and the median RT of the remaining trials was computed in each of these cells. If one cell’s median RT lies outside ± 3 SD (Standard Deviation) from the subject’s average of median RTs computed across the 8 cells, the subject was excluded.

We emphasize that only correct responses were considered in this analysis.

Outcome neutral criteria: First of all, a significant difference of RT between the informative trials and uninformative trials in the pre-exposure phase was a prerequisite to demonstrate that, at the group level, our attention task readily engaged the orienting of endogenous attention. For that purpose, a 2-way rmANOVA was performed on RT of the pre-exposure phases only, with cue type as 2-level factor (informative/uninformative) and exposure as 3-level factor (control, leftward and rightward adaptation). The critical outcome neutral criterion was a main cue type effect and an absence of significant interaction between cue type and exposure factors, which would allow us to demonstrate a significant difference of RT during pre-exposure between informative trials and uninformative trials, irrespective of the exposure session.

Statistical analysis: For this analysis, the dependent variable was the subjects’ cue benefit on discrimination RT, which was computed as follows:

$$Cue\,benefi{t}_{exposureofinterest}=\frac{R{T}_{Uninformative}-R{T}_{Informative}}{R{T}_{Informative}}$$

This dependent variable was averaged in each of the 12 experimental cells defined from the factors of the following rmANOVA, and then submitted to this rmANOVA, with the target hemifield (left or right), the phase (pre- or post-exposure) and the exposure (leftward adaptation, rightward adaptation, or control) as within-factors.

Post Hoc analyses of significant interaction was performed using paired Student t-tests separately for each of the three exposure conditions. The three p-values were then FDR corrected.

After highlighting an effect of leftward adaptation on cue benefit in both hemifields in Experiment 2 (see section Experiment 2 section of the attentional task in the results), we wanted to address whether the beneficial effect of SA on orienting of attention was due to a specific change in RT for informative trials relative to uninformative trials. We thus performed, for the leftward adaptation exposure and separately for informative and uninformative trials, a one-way rmANOVA on median RT with phase (pre- or post-exposure) as the within subject factor.

Finally, we sought for a correlation (Pearson’s product-moment correlation) between the after-effect of leftward saccades adaptation (see formulae above) and the relative change of cue benefit between the pre- and the post-exposure of leftward adaptation, calculated as follow:

$$Relative\,Chang{e}_{Cuebenefit}=\frac{Cue\,benefi{t}_{post-exposure}-Cue\,benefi{t}_{pre-expsoure}}{Cue\,benefi{t}_{pre-expsoure}}$$

## Results

### Pre- and post-exposure saccadic tasks

**Figure 4:**
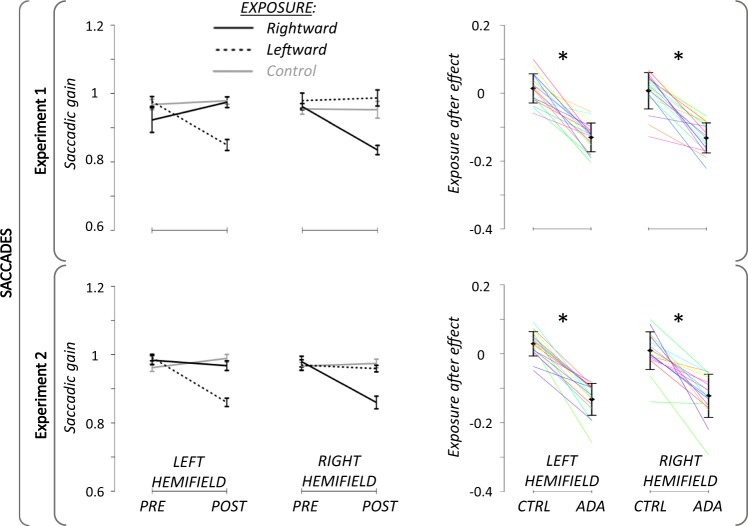
Pre- and Post-exposure saccadic task results. Left panels: Group mean (+/−SD) of saccadic gain for Experiment 1 (upper panel) and Experiment 2 (lower panel). Black lines: rightward adaptation exposure; Black dotted: leftward adaptation exposure; Grey lines: control exposure. Right panels: Individual percent gain changes between the pre- and the post-exposure tasks (after-effect) for Experiment 1 (upper panel) and for Experiment 2 (lower panel). Only data from the adapted hemifield are shown for adaptation exposures (ADA), i.e. left or right hemifield for adaptation exposure of leftward and rightward saccades, respectively; and values of the control exposure (CTRL) are plotted for each corresponding hemifield. Solid black lines represent group mean (+/− SD) and colored lines stand for individual values. *: p-value < 0.05.

After rejection of subjects and trials following the above mentioned criteria (see preprocessing section for details), the average number of analysed trials per condition was 13.6 +/− 1.3 SD in Experiment 1 and 12.4 +/−1.7 SD in Experiment 2. The mean saccadic gain in pre- and post-exposure, as well as the individual and mean adaptation after-effect, are illustrated in Fig. 4. As it was a pre-requisit (see subjects section), all subjects of each experiment showed in the adaptation sessions a significant decrease of the saccadic gain for target presented in the adapted hemifield, in the post-exposure as compared to the pre-exposure, thus having a significant after-effect due to SA (Fig. 4, right panel). Moreover, as seen in Fig. 4 (left panel), this decrease was not seen in the opposite, unadapted, hemifield, whether for the leftward or rightward exposure. In addition, the amounts of adaptation in the adapted hemifields did not differ between the leftward and rightward exposures, both for Experiment 1 (Cohen’s *d = *0.04; t_17_ = 0.14; P = 0.89) and Experiment 2 (Cohen’s *d* = 0.05; t_17_ = 0.21; P = 0.84). Finally, no gain change in either hemifield took place in the control exposure.

### Attention task

#### Experiment 1 (target at 7.5°)

**Figure 5:**
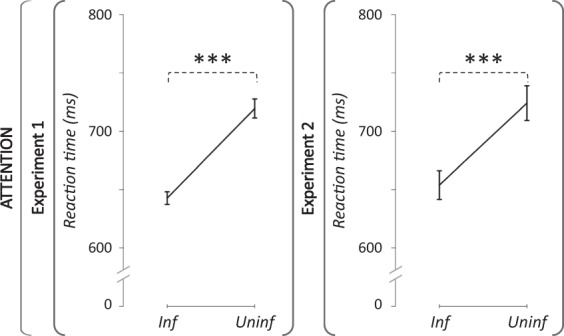
Effect of cue type on reaction time in the pre-exposure attention task. Group mean (+/−SD) of median reaction times (ms) in Experiment 1 (left panel) and in Experiment 2 (right panel). A general effect of cue type was disclosed by the decrease of RT for informative as compared to uninformative trials. ***: p-value < 0.001.

Outcome neutral criteria: After rejection of subjects and trials following the above mentionned criteria, the average number of analysed trials per condition was 58.9 +/− 5.6 SD (see preprocessing section for details). The rmAnova of outcome neutral criteria on the pre-exposure RT revealed a significant main effect of the cue type (partial η² = 0.81; F_(1,17)_ = 74.15; P = 1.32e^−7^; achieved power = 1; Fig. 5, left panel). The main effect of the exposure was not significant (partial η² = 0.01; F_(2,34)_ = 0.17; P = 0.85), nor the interaction between exposure and cue type (partial η² = 0.01; F_(2,34)_ = 0.28; P = 0.76). Therefore, our attention task did engage the orienting of attention during the pre-exposure phase, and did so similarly in the three sessions.

**Figure 6:**
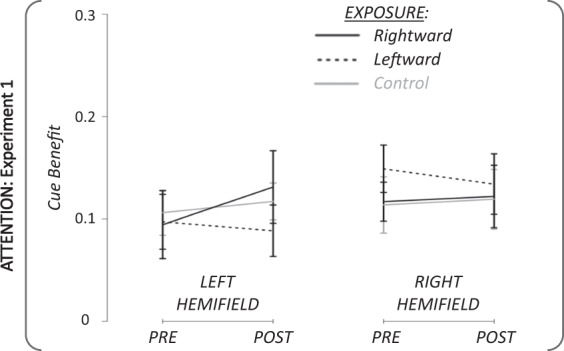
Pre- and Post-exposure attention results in Experiment 1. Group mean (+/−SD) of cue benefit for the pre- and the post-exposure phases in the two hemifields of target presentation. Black lines: rightward adaptation exposure; Black dotted lines: leftward adaptation exposure; Grey lines: control exposure.

Statistical analysis: The performance in the attention task was evaluated by computing the cue benefit of subjects’ (Fig. 6). Submitting cue benefit to a rmANOVA with the factors exposure × phase × target hemifield revealed no significant main effect and no significant double nor triple interaction (all P > 0. 32). Therefore, no further analysis was performed. In summary, no significant effect of saccadic adaptation on attention performance could be revealed when the target was presented at 7.5°.

#### Experiment 2 (target at 3°)

Outcome neutral criteria: After rejection of subjects and trials following the above mentionned criteria, the average number of analysed trials per condition was 57.0 +/− 7.4 SD (see preprocessing section for details). The rmAnova of outcome neutral criteria on the pre-exposure RT revealed a significant main effect of the cue type (partial η² = 0.64; F_(1,17)_ = 30.81; P = 3.52e^−5^; achieved power = 1; Fig. 5, right panel). The main effect of the exposure was not significant (partial η² = 0.03; F_(2,34)_ = 0.44; P = 0.65), nor the interaction between exposure and cue type (partial η² = 0.05; F_(2,34)_ = 0.81; P = 0.45). Thus, as for Experiment 1, the attention task in Experiment 2 engaged the orienting of attention during the pre-exposure phase, and did so similarly in the three sessions.

**Figure 7:**
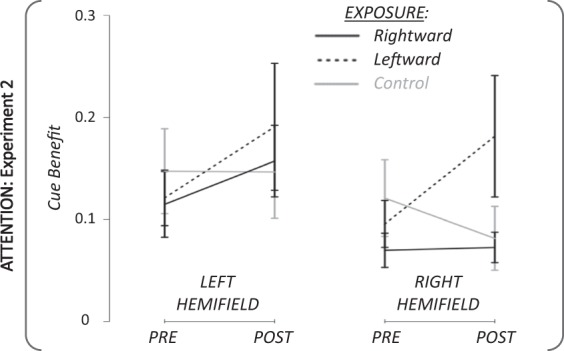
Pre- and Post-exposure attention results in Experiment 2. Group mean (+/−SD) of cue benefit for the pre- and the post-exposure phases in the two hemifields of target presentation. Black lines: rightward adaptation exposure; Black dotted: leftward adaptation exposure; Grey lines: control exposure.

Statistical analysis: As for Experiment 1, the performance in the attention task of Experiment 2 was evaluated by computing the subjects’ cue benefit (Fig. 7). The 3-factor rmANOVA (exposure × phase × target hemifield) revealed no significant main effect (phase: partial η² = 0.10; F_(1,17)_ = 1.98; P = 0. 18; target hemifield: partial η² = 0.11; F_(1,17)_ = 2.00; P = 0.18; exposure: partial η² = 0.49; F_(2,34)_ = 0.07; P = 0.29). The following interactions were not significant: double interactions (exposure × target hemifield: partial η² = 0.09; F_(2,34)_ = 1.59; P = 0.22; phase x target hemifield: partial η² = 0.05; F_(2,34)_ = 0.95; P = 0.34), and the triple interaction (exposure × phase × target hemifield: partial η² = 0.05; F_(2,34)_ = 0.92; P = 0.41). However, the double interaction exposure × phase was significant (partial η² = 0.18; F_(2,34)_ = 3.76; P = 0.03; achieved power >99%).

Post-hoc paired Student t-tests revealed that, irrespective of hemifield, the differences between the pre- and the post-exposure phases for the control exposure and for the rightward adaptation exposure did not reach significance (t_(35)_ = 0.92; P = 0.36; t_(35)_ = −1.53; P = 0.13; respectively). In contrast, the exposure to leftward adaptation induced a significant difference between the pre- and the post-exposure, yielding an increased cue benefit in both hemifields (from 0.11 to 0.19; 95 CI mean difference = [−0.14; −0.02]) after SA (t_(35)_ = −2.56; P = 0.015 (FDR- corrected P = 0.045); Cohen’s *d* = 0.40).

To identify the origin of the significant exposure x phase interaction, we further submitted RT to 2 one-way rmANOVAs, separately for informative or uninformative trials. The rmANOVA on informative trials revealed a nearly significant effect of the phase (partial η² = 0.20; F_(1,17)_ = 4.16; P = 0.057), whereas the rmANOVA applied to uninformative trials did not (partial η² = 0.06; F_(1,17)_ = 1.00; P = 0.33). Therefore, the boosting effect of adaptation on discrimination speed is mainly observed for informative trials, i.e. trials that most strongly elicited an endogenous orienting of attention.

Concerning the link between the change in the left saccadic gain and the change in the cue benefit (Figure in Supplementary), after leftward adaptation, we did not highlight a significant correlation (r_(35)_ = 1.30; P = 0.21).

We then computed the post-saccadic target error during the exposure to leftward SA. Mean values are plotted in Table 1 separately for each block of adaptation exposure and for the 4 blocks pooled together.

**Table 1:** Mean and standard deviation (SD) across subjects of post-saccadic error in the exposure to backward adaptation of leftward saccades.

	Block 1	Block 2	Block 3	Block 4	All Blocks
Mean	0.42	0.99	1.64	1.35	1.09
SD	0.34	0.48	0.57	0.61	0.49

Table 2 summarizes the results of the correlation between this post-saccadic error parameter and the cue benefit measured during the post-exposure discrimination task. None of the analyses revealed any significant correlation, whether computed separately for the 4 blocks or for all blocks together.

**Table 2:** Rho coefficients (degree of freedom = 16) and p-values of the correlations between post-saccadic error and cue benefit.

	Block 1	Block 2	Block 3	Block 4	All Blocks
r_(16)_	−0.29	−0.34	−0.18	−0.10	−0.20
p-value	0.24	0.17	0.47	0.69	0.43

Finally we assessed as a potential attentional marker the preparation time of voluntary saccades by measuring the fixation time between the first and second saccades: the overall median fixation time is 271 ms with an interquartile range (IQR) of 122 ms (see details in Table 3). The rmANOVA did not reveal any significant main effect (saccade direction: partial η² = 0.02; F_(1,17)_ = 0.30; P = 0.59; phase: partial η² = 0.02; F_(1,17)_ = 0.30; P = 0.59) nor an interaction between the two factors (partial η² = 0.002; F_(1,17)_ = 0.03; P = 0.89). Therefore, no significant effect of SA on the fixation time preceding voluntary saccades could be demonstrated.

**Table 3:** Median and interquartile range (IQR) fixation time across subjects for the pre- and post-phase of the exposure to backward adaptation of leftward saccades and of rightward saccades.

	Leftward saccades		Rightward saccades	
	Pre-exposure	Post-exposure	Pre-exposure	Post-exposure
Median (ms)	269.75	272.75	275.75	266
IQR (ms)	117.25	138.5	97.125	117.75

In summary, the adaptation of leftward saccades resulted in a significant increase of attention performance when the target was presented at 3° in both the adapted and unadapted hemified, but without significant relationship with individual variations of the level of adaptation or of the amount of post-saccadic target error.

## Discussion

The present study questioned the link between the oculomotor and visuospatial attention systems, by testing the effect of sensorimotor plasticity of VS on covert endogenous orienting of attention. Based on a within-subjects comparison between leftward adaptation, rightward adaptation, and control exposure, we found the cueing effect on discrimination RT to increase specifically after leftward adaptation for discrimination stimuli at 3° in either (adapted or unadapted) hemifields. This boosting effect of SA was mainly related to a decreased RT for informative trials, i.e. those that elicited an endogenous orienting of attention. No effect was observed on saccade prepration time. These results demonstrate for the first time a boosting effect of oculomotor plasticity on endogenous orienting of attention in healthy humans, deepening our knowledge of saccadic adaptation mechanisms and providing evidence for shared neuronal representations for eye movements and visuospatial attention.

As mentioned in the Introduction, a coupling between SA and covert shifts of attention has been reported only once at the behavioral level in a previous study from our lab^13^. However, contrary to the Posner-like paradigm used here, the detection task Habchi and colleagues^13^ used could not entirely distinguish attention orienting from other potential cognitive or motor components. In addition, they investigated exogenous attention orienting. Here we decided instead to focus on the voluntary/endogenous modality, because it has been suggested to refute the premotor theory of attention^15^. The present demonstration of a coupling in this latter modality therefore provides a new piece of empirical argument in this debate.

Interestingly, despite these differences, in both Habchi and colleagues’ study and ours, the coupling was observed only after adaptation of leftward saccades. They interpreted this saccade direction specificity as resulting from the known dominance of the right hemisphere in controlling exogenous attention^4^, without making any assumption of hemispheric laterality for saccadic adaptation, which is completely unknown. Indeed, in the relevant fMRI literature, only cortical BOLD modulations after leftward SA have been investigated so far^8,12,26^. A right dominance interpretation of the saccade-direction specific coupling demonstrated here for the voluntary/endogenous modality is not straightforward in this framework. However, TMS studies have suggested that, although both left and right IPS play a role in voluntary orienting visuospatial attention, the right hemisphere has a dominant contribution. Caposto and colleagues^27,28^ reported that the disruption of the right IPS, and not the left IPS nor the right FEF, led to a bilateral alpha band synchronization in the occipito-parietal cortex and therefore to a decreased efficiency of target processing in both hemifields. Indeed, alpha synchronization and desynchronization are known to index visual perception performance: the lower alpha power the better the performances e.g.^29–32^. The impact of the right IPS disruption was also observed in two studies^33,34^ using concurrent TMS/fMRI in which stimulation of right but not left posterior parietal cortex caused changes of fMRI activity bilaterally in the occipital lobe. Thus, the presently demonstrated effect of adaptation of leftward, but not rightward, VS fits in the framework of a right hemispheric dominance in visuospatial attention. In addition, the benefit in the two hemifields that we found in the attention task is consistent with the above mentioned TMS studies. Indeed, it can be postulated that SA of leftward saccades, contrary to the disrupting effect of TMS, increases brain excitability in the right IPS and therefore modulates neural excitability in the occipital cortex bilaterally.

Other previous investigations of the link between SA and visuospatial attention have all focused on the so-called pre-saccadic shift of attention, corresponding to an enhanced perception which automatically occurs at the saccade target location just before saccade initiation^35^. These studies have shown that after saccadic adaptation, the spatial locus of highest perceptual performance remains coupled with the saccade endpoint, not to the visual target^36–38^ (but see^39^). In line with the premotor theory of attention^5^, this observation reflects an adaptation-related change of a prediction of saccadic commands, which is also consistent with the proposal that oculomotor efference copy is modified after adaptation^38^.

The present findings clearly point to a new oculomotor plasticity-visuospatial attention coupling as compared to the studies mentioned above. First, the lack of significant correlation between the adaptation rate and the cue benefit boost does not illustrate the metrical relationship found in previous studies between saccade size and endpoint of pre-saccadic attention shift^40^. Together with the specificity to a 3° eccentricity, this observation suggests an all-or-none effect restrained to the peri-foveal part of the visual field. Second, we demonstrated an effect of SA on covert shifts of attention, unrelated to any oculomotor preparation, as subjects always kept central fixation throughout the attention tasks. Thus, possible changes of oculomotor efference copy are unlikely to play any role in our experiments. Furthermore, the discrimination performance did not change for a target at 7.5°, i.e. the eccentricity which matched best the adapted saccade endpoint. Thus, the coupling we report is not related to the new metric of the adapted saccade, and not to the adaptation field^41,42^. Instead the boosting effect was actually found at the eccentricity of 3° which corresponds to the size of the target intra-saccadic step (ISS) eliciting SA. This raises the interesting possibility that it is the systematic exposure to the error signal driving SA, rather than the oculomotor changes related to SA itself, which drives the changes in covert attention. Recall however, that the same target jump and error signal were induced during the control exposure, but 805 ms after the saccade, a delay which prevented SA to be elicited. Moreover, our analyses failed to disclose any significant correlation between the post-saccadic error experienced during the leftward adaptation exposure and the cue benefit measured during the post-exposure discrimination task. Therefore the correspondence between the size of ISS and the eccentricity for which the effect was found could be a mere coincidence. Further experiments would be required to test this possibility. For example, one could induce adaptation of larger saccades with larger target jumps and test whether the eccentricity where the boosting effect occurs changes accordingly or remains in the peri-foveal part of the visual field.

Another possible explanation of this limitation to the peri-foveal part of the visual field is a SA-induced compression of represented visual space (in case of backward adaptation) that would shift the representation of visual stimuli toward the center of gaze. Indeed, Zimmermann and Lappe^43,44^ showed that SA induces a shift of the subjectively-perceived location of objects flashed before a saccade or during fixation, suggesting that spatial visual representations are shaped by oculomotor planning^45,46^. Consequently, when subjects have to localize (Zimmermann and Lappe’s) or discriminate (current study) such peri-foveal stimuli, they would both underestimate the targets eccentricity and discriminate them with a faster reaction time. The functional coupling between adaptation and attention, highlighted by the present results, strongly suggests that the corresponding neural substrates overlapping at the macroscopic level (see introduction^3,8,9^) actually host neuronal population co-activated for saccades and attention. Although neuronal recordings in the monkey posterior parietal cortex have provided evidence for distinct neuronal populations for orienting of attention and saccadic eye movements^47^, other studies have suggested that the monkey LIP hosts priority maps used both by attention and eye movements to select targets^48^. Therefore, we believe that SA acts on such ‘common priority maps’, thereby transferring to covert attention mechanisms. Common priority maps for attention and eye movements may have been implemented in the course of natural selection because sharing neural substrates for cognitive functions is advantageous in terms of neural resource. Accordingly, as mentioned above, we propose that the boosting effect would emerge from an SA-induced increase of top-down signals from the right parietal cortex to the visual cortex of both hemispheres. Indeed, there is increasing evidence that adaptation of leftward saccades relies on metabolic activation in the right IPS^8,9,11,12^. Second, during an endogenous attentional orienting task, the right IPS send top-down signals to the visual cortices in both hemispheres to modulate their excitability and therefore their readiness to process an upcoming stimulus (see^4^ for review). To account for the observed boosting effect restricted to +/−3° eccentric targets, we further suggest that the increased activity of the IPS is centered on the fovea, which is in accordance with the oculocentric representation of visual space in the posterior parietal cortex^49^. The boosting of attention we specifically observed after leftward SA for targets flanking the fovea bilaterally seems to be related to the dominant role of the right IPS in the control of visual attention and to its properties in representing the visual space. This specificity speaks for a functional link between adaptation of voluntary saccades and endogenous visuospatial attention based on the brain substrates common to these processes, rather than on a general increase in brain excitability after SA.

The hypothesis of shared neural resource between adaptation and attention predicts the existence of another functional coupling, opposite to that reported here, i.e. from attention to saccadic adaptation. Indeed, some studies have suggested that attention shifts affect SA. Flashing in the vicinity of a stationary saccade target a stimulus attracting exogenous attention, a perceptual target^50^ or a salient visual distractor^51^, is sufficient to induce SA. Further, McFadden *et al*.^52^ showed that it is possible to adapt the exogenous shift of attention by ‘stepping the attentional target’ during a covert attentional task, and that such ‘adapted attention’ transferred to saccades. Finally, SA efficiency has been shown to increase with attentional load^14^. The hypothesis of shared neural substrates between adaptation and attention also predicts that some neural changes related to SA can be detected in the attentional task performed immediately after, akin to the change of gamma band activity we could disclose recently, albeit in the exogenous/reactive modality^11^. A similar magnetoencephalographic study will be required to disclose whether the coupling between adaptation of voluntary saccades and endogenous attention is subtended by an increased brain activity, reflected in the gamma band, in the region of the right IPS of the dorsal attention system.

Taken together, this study highlights a functional coupling between adaptation of voluntary saccades and endogenous visuospatial attention. This finding provides deeper insight into the role of the motor system in the updating of visual space representations, and leads toward promising rehabilitation procedure for patients with visuospatial disorders.

## Supplementary information

Supplementary material
