# Supplementary material for: Inducing oculomotor plasticity to disclose the functional link between voluntary saccades and endogenous attention deployed perifoveally
Source: Sci Rep. 2019 Nov 28;9:17770. doi: 10.1038/s41598-019-54256-1 (PMC6882914; doi:10.1038/s41598-019-54256-1)
Supplement: Supplementary file 1 — Supplementary material [file 41598_2019_54256_MOESM1_ESM.docx]

*TITLE PAGE*

Title: “Inducing oculomotor plasticity to discloses the functional link between voluntary saccades and endogenous attention deployed perifoveally”

Judith Nicolas^1,2,3^, Aurélie Bidet-Caulet^2,3^, and Denis Pélisson^1,3^

^1^Integrative Multisensory Perception Action & Cognition Team (ImpAct), INSERM U1028, CNRS UMR5292, Lyon Neuroscience Research Center (CRNL), 69000 Lyon, France

^2^Brain Dynamics and Cognition (Dycog Team), INSERM U1028, CNRS UMR5292, Lyon Neuroscience Research Center (CRNL), 69000 Lyon, France

^3^University Claude Bernard Lyon 1, Université de Lyon, 69000 Lyon, France

Corresponding author: Judith Nicolas, INSERM U1028, CNRS UMR5292, Lyon Neuroscience Research Center, ImpAct Team, 16 Avenue Doyen Lépine 69500 Bron, France. Email: [judith.nicolas@inserm.fr](mailto:judith.nicolas@inserm.fr) ;

# SUPPLEMENTARY METHODS

Power analysis. The number of subjects was determined from the following power analysis performed through the G*Power software^1^. As stated in Introduction, we are aware of only one previous study testing the coupling between SA and visuospatial attention^2^. This study disclosed that after adaptation of RS in the left hemifield, the performance in a visual detection task improved in the left hemifield. This was revealed by a significant 3-level interaction in a repeated measures ANOVA (rmANOVA) with subjects as the repeated measure, the target hemifield (left or right), the phase (pre- or post-exposure) and the exposure (leftward adaptation, rightward adaptation, or control) as within-factors. We have computed the effect size of this interaction and found a value of 0.41 (Sum of Square of the numerator = 485.7; Sum of Square of the denominator = 695.8), resulting in an achieved power larger than 99% according to the G*Power software. However, since the present study will address another modality (i.e. voluntary saccades and endogenous attention), we decided to reduce this effect size to a conservative level of 0.2.

Given this medium effect size (f = 0.2), we found that 14 subjects are required for our interaction of interest between the 3 within-subjects to reach a power of 95.3% when assuming an average correlation coefficient of r = 0.85 between repeated measures and a nonsphericity correction of ε=0.5 (see below). To counterbalance the six possible testing orders in the sessions included in our design, we decided to increase for each experiment this number up to 18 subjects, reaching a power of 98.9%.

The power analysis is also based on a pilot study we performed to find the best Stimulus Onset Asynchrony (SOA) for our endogenous task. The SOA, the duration between the cue appearance and the target appearance, is the time allocated to endogenous attention to shift and develop. Our pilot study consisted in testing the attention task described in the main text (see Attention task: visual discrimination in the section Apparatus, stimuli and procedure of the MATERIALS AND METHODS) for 3 different SOAs (602 ms / 850 ms and 1106 ms), each SOA condition being evaluated in a separate session comprising 3 blocks of 52 trials. These pilot data (RT of discrimination response) allowed us to establish a main effect of cue type regardless of the SOA duration (effect size = 0.88; F(1,3) = 22.3384; p-value = 0.0179). No main effect of SOA nor interaction between SOA and cue type was significant. They also allowed us, using the Mauchly’s test for sphericity, to check that the sphericity of the 3-level factor SOA is respected (W=0.27; p-value = 0.279). Finally, these data allowed us to determine the correlation between repeated measures: the correlation of discrimination RT between the three SOA conditions (602 ms versus 850 ms, 602 ms versus 1106 ms, and 850 versus 1106 ms), using Pearson's product moment correlation coefficient tests, turned out to be highly significant in all three cases (p-values < 10-5 and r-values > 0.85). We therefore set the correlation between repeated measures at r=0.85.

Staircase procedure. Before the pre-exposure phase of the first session, a staircase procedure allowed us to determine the gabor patches contrast, separately for each subject and each hemifield. A transformed 3-down 1-up procedure was used^3,4^. For each hemifield, one staircase starting with a median contrast (50%) was run, leading to 2 interleaved staircases. A staircase trial consisted of the same sequence of events as in the attention task except that all cues were uninformative and the time between the cue and the target reduced to 500 ms. The procedure ended as soon as it reached 15 reversals in each hemifield. The final contrast was determined separately for each hemifield by averaging the 4 last reversal values. Before the staircase procedure, subjects performed at least 25 trials of practice with a gabor patch of 100% contrast (20 trials plus five consecutive correct trials). The following instructions were displayed at the beginning of the practice block: “clockwise or anti-clockwise?; clockwise => push; anti-clockwise => pull”. The experimenter displayed an example of both types of trial (informative and uninformative) and could repeat the examples until the subject got familiar with them.

Instructions and feedback. After the staircase procedure was completed, and when the subject was ready, the attention task started by first displaying on the screen instructions on the use of the cue. These instructions were read as follows (translation form French): “Now, you will have arrows to help you; RIGHT arrows => RIGHT target; LEFT arrows => LEFT target; OUTWARD arrows => RIGHT or LEFT; INWARD arrows => DO NOT ANSWER”. Then, we displayed instructions on how feedback about their discrimination performance are provided after each block of trials, namely by means of a gauge filling up or down depending on subject’s performance (“if you use the arrow well enough to be fast and accurate, the gauge will fill up otherwise it will empty”). At the beginning of each session, this 10 graduations gauge was initially filled to the fourth graduation. At the end of each block the gauge was presented again but filled to a new graduation according to the subjects’ score during the block: one graduation was gained if they were either faster or more accurate in informative trials than in uninformative trials, two graduations was gained when the two criteria were met, and conversely, one graduation was lost if either subjects’ median performance was slower and less accurate in informative trials than in uninformative trials or if their overall performance is less than 70% correct. Increases or decreases of gauge levels were emphasized by a green or red filling color, respectively. In addition, changes of gauge level were accompanied by the following sentences: “Be careful, you need to better use the arrows” (one graduation down), “Good, but you can still better use the arrows” (one graduation up); “Bravo, keep on using the arrows this way!” (two graduations up). This procedure aimed to reinforce the use of the cue without favoring either speed or accuracy. Finally, a sentence was displayed requesting the subject to signal when she/he was ready to start the next block. These written feedbacks and instructions are intended to avoid non-standardized oral feedback from the experimenter who was not blind regarding the type of session (leftward adaptation, rightward adaptation, control).

# SUPPLEMENTARY RESULT


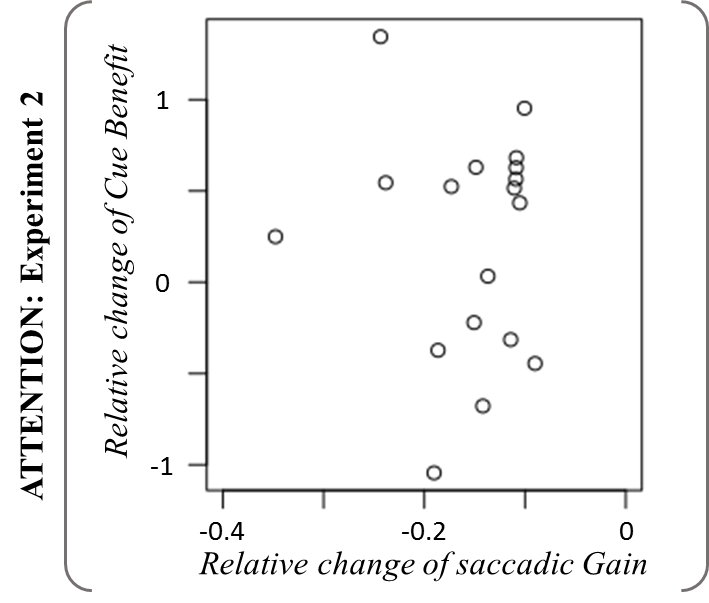


***Supplementary Figure 1: Relative change of cue benefit (irrespective of hemifield) in function of relative change of saccadic gain in the leftward adaptation exposure.*** *Points represent individual data.*

##### SUPPLEMENTARY REFERENCES

1. Faul, F., Erdfelder, E., Lang, A.-G. & Buchner, A. G* Power 3: A flexible statistical power analysis program for the social, behavioral, and biomedical sciences. Behavior research methods 39, 175–191 (2007).

2. Habchi, O. et al. Deployment of spatial attention without moving the eyes is boosted by oculomotor adaptation. Frontiers in Human Neuroscience 9, (2015).

3. Levitt, H. Transformed Up‐Down Methods in Psychoacoustics. The Journal of the Acoustical Society of America 49, 467–477 (1971).

4. Leek, M. R. Adaptive procedures in psychophysical research. Perception & psychophysics 63, 1279–1292 (2001).
